# Does Cortical Inhibition Explain the Correlation Between Bistable Perception Paradigms?

**DOI:** 10.1177/20416695211020018

**Published:** 2021-05-26

**Authors:** Abhilasha R. Jagtap, Jan W. Brascamp

**Affiliations:** Department of Psychology, 3078 Michigan State University, East Lansing, United States

**Keywords:** binocular rivalry, bistable moving plaids, center-surround suppression, cortical inhibition, individual differences

## Abstract

When observers view a perceptually bistable stimulus, their perception changes stochastically. Various studies have shown across-observer correlations in the percept durations for different bistable stimuli including binocular rivalry stimuli and bistable moving plaids. Previous work on binocular rivalry posits that neural inhibition in the visual hierarchy is a factor involved in the perceptual fluctuations in that paradigm. Here, in order to investigate whether between-observer variability in cortical inhibition underlies correlated percept durations between binocular rivalry and bistable moving plaid perception, we used center-surround suppression as a behavioral measure of cortical inhibition. We recruited 217 participants in a test battery that included bistable perception paradigms as well as a center-surround suppression paradigm. While we were able to successfully replicate the correlations between binocular rivalry and bistable moving plaid perception, we did not find a correlation between center-surround suppression strength and percept durations for any form of bistable perception. Moreover, the results from a mediation analysis indicate that center-surround suppression is not the mediating factor in the correlation between binocular rivalry and bistable moving plaids. These results do not support the idea that cortical inhibition can explain the between-observer correlation in mean percept duration between binocular rivalry and bistable moving plaid perception.

## Introduction

**Figure 1.**
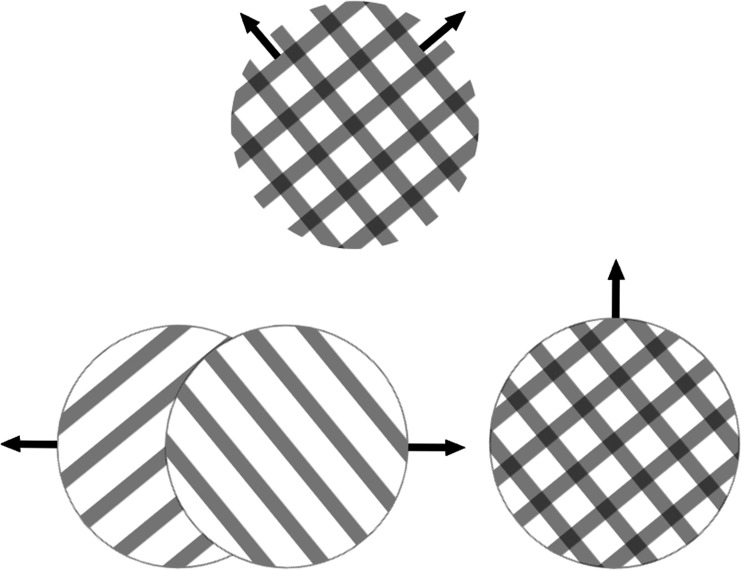
Bistable moving plaids. The plaid pattern that is schematically depicted at the top was shown during the experiment. There are two compatible percepts: one where the two gratings seem to slide over each other and a second one in which the pattern seems to be moving coherently in one direction.

Binocular rivalry is the stochastic fluctuation of perception that occurs when interocularly conflicting visual information is presented to the two eyes (Blake & Logothetis, 2002) such that the two eyes’ images are perceived in turn. While this phenomenon has been widely studied, the mechanisms underlying these perceptual fluctuations are not clear (Kang & Blake, 2010; Logothetis et al., 1996). Investigating which other bistable phenomena are correlated with binocular rivalry in terms of the time course of their perceptual fluctuations can increase scientific understanding of binocular rivalry by identifying common factors that drive the perceptual cycles across multiple paradigms. Previous studies (Brascamp et al., 2019; Cao et al., 2018; Sheppard & Pettigrew, 2006) have indicated that the rate at which perception switches (and, relatedly, the average duration of individual percepts) is strongly correlated between binocular rivalry and bistable plaid perception (Figure 1), suggesting a shared mechanism underlying both phenomena. The next logical question is as follows: what is this mechanism?

Theories about binocular rivalry posit that neural inhibition at multiple levels in the visual hierarchy could be responsible for suppressing the representation of one of the eyes’ images during binocular rivalry, thus playing an important role in the perceptual cycle (Tong et al., 2006; van Loon et al., 2013). Therefore, a change in the excitatory–inhibitory balance in the cortical areas (especially visual cortex) has been suggested to influence the perceptual fluctuations of binocular rivalry. Consistent with this idea, evidence from previous studies (McKendrick et al., 2019; van Loon et al., 2013, although see Sandberg et al., 2016) indicates that as the concentration of gamma aminobutyric acid (GABA) or (the brain’s main inhibitory neurotransmitter) in the visual cortex increases, there is a decrease in perceptual switch rate during binocular rivalry. Furthermore, a study on older adults (Pitchaimuthu et al., 2017) reported that the concentration of GABA is significantly greater in older adults as compared with younger adults and that older adults report fewer perceptual switches as compared with younger adults. In addition, disorders like schizophrenia and autism spectrum disorder (ASD) are linked to a disturbance in the excitatory–inhibitory balance (Spiegel et al., 2019; Yoon et al., 2010), and also with altered binocular rivalry dynamics. At least two studies have found that patients suffering from schizophrenia had lower switch rates for binocular rivalry as compared with their healthy counterparts (Xiao et al., 2018; Ye et al., 2019). Similarly, a study of binocular rivalry in ASD observed that there is a significant reduction in the alternation rates in this population (Spiegel et al., 2019). Yet another study found similar results and reported that an inverse relationship between the concentration of cortical GABA and the proportion of perceptual suppression during rivalry, which is observed in healthy controls, is absent in ASD patients, thus suggesting that the disturbance in excitatory–inhibitory balance in the autistic brains leads to a reduced effect of GABA on perceptual suppression (Robertson et al., 2016).

Putting these pieces of evidence together, this study aims to investigate whether a dependence on cortical inhibition may be the common factor that causes a correlation between the dynamics of binocular rivalry and bistable plaid perception that is indicated by existing work.

If this is true, then one might expect the dynamics of these two bistable perceptual phenomena to show correlations with other perceptual phenomena linked to inhibition and one might expect these correlations to (partly) account for the observed relation between binocular rivalry and bistable plaid perception. Here, we focus on one such phenomenon that is commonly linked to inhibition, namely, surround suppression: A reduction in the apparent contrast of a central stimulus as a result of the presence of a surrounding pattern (Cannon & Fullenkamp, 1993; Chubb et al., 1989; Xing & Heeger, 2000). A reduced perceptual strength of surround suppression has been linked to a reduction in cortical GABA concentrations in the same patient populations also mentioned earlier in the context of altered binocular rivalry perception (Flevaris & Murray, 2015; Serrano-Pedraza et al., 2014). Additional evidence from a recent study (McKendrick et al., 2019) indicates that there is, indeed, a positive correlation between the perceptual strength of surround suppression and percept durations for binocular rivalry, further encouraging the hypothesis that inhibition could be an underlying factor, perhaps mediated by GABA concentration, that explains the correlation in percept durations between binocular rivalry and bistable plaid perception.

**Figure 2.**
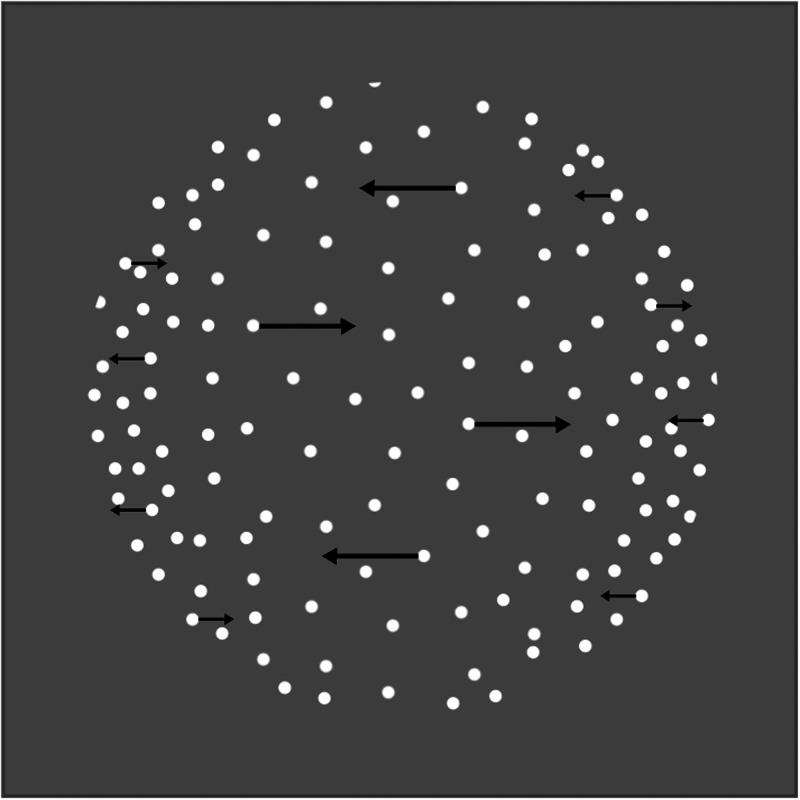
Rotating sphere. This is a schematic of the rotating sphere stimulus, which is composed of dots moving horizontally and confined to a circular area. The speed profile of the dots, as well as dot density, was varied as a function of position within the circle, consistent with an orthographic projection of a dotted, transparent sphere. For example, dot speed was highest near the center of the circle and lowest near the outer edge of the circle. This gave rise to the perception of a rotating sphere, but the direction of rotation was ambiguous.

As outlined earlier, we hypothesize that the previously reported correlation between percept durations of binocular rivalry and bistable plaid perception can be accounted for, in part, by across-observer variance in the perceptual strength of center-surround suppression. We test this hypothesis with the help of a test battery which includes binocular rivalry, bistable moving plaids, and a (non bistable) center-surround suppression task. In addition, we include a bistable structure-from-motion stimulus (Figure 2). While correlated perceptual dynamics have been consistently observed for the combination of binocular rivalry and bistable plaid perception (Brascamp et al., 2019; Cao et al., 2018; Sheppard & Pettigrew, 2006), this picture is less consistent for other combinations of bistable stimuli, including the combination of binocular rivalry and bistable structure-from-motion (Brascamp et al., 2018; Cao et al., 2018 show no significant correlation, whereas Steinwurzel et al., 2020 show a positive correlation between switch rates in these two paradigms). Adding a structure-from-motion stimulus to our battery, therefore, allowed us to reexamine any correlation with the other bistable phenomena and, if it is observed, to test whether it is (partly) accounted for by variance in the strength of surround suppression.

## Methods

### Participants

We recruited 217 healthy participants, aged between 18 and 25 years, from the psychology subject pool of Michigan State University. Of these 217 participants, 113 (34 males) were recruited in one semester and 104 (18 males) in the following semester. The study was conducted over three sessions in one semester and over two sessions in the other semester (the sessions also included experiments unrelated to the present work; see later). The task order within each session was fixed, but the order of sessions was counterbalanced across participants in both the semesters (in one semester involving three sessions, the binocular rivalry task and bistable moving plaids task were run in a single session and the other two paradigms were each run in one of the remaining sessions whereas in the other semester, the center-surround suppression task was run along with the binocular rivalry task and the bistable moving plaids task in one session, and structure-from-motion task was run in a separate session). Not all recruited participants showed up to (all of) the testing sessions, and data were collected from a total of 196 participants for binocular rivalry, 195 participants for bistable moving plaids, 192 for structure-from-motion, and 196 for the surround suppression task. For each task, some further participants were dropped after preliminary analysis of the data (see later for exclusion criteria). The final sample for which the data were fully analyzed (beyond preliminary analysis; details in “Data Analysis” section) consisted of 171 participants for binocular rivalry, 181 participants for bistable moving plaids, 178 participants for structure-from-motion, and 151 participants for the surround suppression task.

The study was approved by the Michigan State University institutional review board and was conducted over the course of two semesters. Written informed consent was obtained from all the participants prior to testing. The testing battery included other tasks not reported here, and this set of tasks differed slightly between the two semesters. The tasks reported here, however, were the same in both cases. Participants fulfilled course requirements through their participation.

### Stimuli and Task

#### Binocular Rivalry

The specifications of this stimulus were identical to those of one of the stimuli (“small grating stimulus”) used in a previous study (Brascamp et al., 2019, p. 3). Sinusoidal gratings (spatial frequency of 2.0 c/dva, Michelson contrast 0.5, mean luminance same as background luminance) were presented within an annular aperture (inner radius 0.37 dva, outer radius of 0.85 dva) on two separate monitors viewed through a mirror stereoscope. Interocular conflict was created by presenting different grating orientations (−45° and +45° from vertical) and grating colors (using either only the monitor’s red channel or only the monitor’s green channel) to each eye. Both eyes were presented with a grating that drifted diagonally upward or downward, at 0.24 dva/s to minimize the formation of negative afterimages. The vertical direction of this movement was always the same in both eyes and was varied from trial to trial.

A mimic condition was also included in which a single stimulus was shown binocularly, as opposed to two gratings being shown dichoptically at the same time. This stimulus could, at different times, consist of either of the grating patterns or a patchwork of both. The latter mimicked a mixture percept of the binocular rivalry stimulus. We mention this mimic condition here because it was part of the same experiment blocks as the binocular rivalry condition, but the data from the mimic condition were not analyzed in the context of this study.

During either condition, observers had to indicate the onset of the red pattern, the onset of the green pattern, and also the onset of mixture percepts where both of the patterns were partly visible using three separate keys on the keyboard. Each block started with a 20-second practice trial with the rivalry stimulus, and the data for this practice trial were not analyzed. Each subsequent trial lasted for 45 seconds, followed by a forced break of 10 seconds (longer if the observer wanted). There were six trials of the binocular rivalry condition and four trials of the mimic condition that were randomly interleaved within the experimental block. Each participant ran a single experiment block.

#### Bistable Moving Plaids

The stimulus specifications for the bistable moving plaids were identical to those used in a previous study (Brascamp et al., 2019). The stimulus consisted of two overlapping square wave gratings oriented at ±26.6° relative to vertical (spatial frequency: 2.0 c/dva; 0.5 Michelson contrast). They were made in grayscale and had a uniform gray background of 35.2 cd/m^2^ (same as the average grating luminance). The gratings shifted diagonally upward at 0.48 dva/s. The luminance of the locations where the two gratings overlapped was adjusted to promote perception of transparency (see later for possible percepts). This was done by first calculating the difference in log space between the luminance of the dark stripes and that of the light stripes of the gratings. The luminance of the intersection area between two bright stripes was then set such that, again in log space, the difference between the intersection luminance and the luminance of the bright stripes was the same. This stimulus gave rise to periods when observers perceived a coherent, solid, diamond pattern moving straight upward, as well as periods when they perceived two transparently superimposed gratings moving in two different directions. A schematic of this stimulus and percepts is presented in Figure 1. The task of the participant was to indicate the onset of either of the aforementioned percepts, as well as the onset of mixture percepts, using three separate keys on the keyboard (the only difference between this task and the one in the 2019 study was this ability to report mixed percepts.). Each trial was 60 seconds long, followed by a 10-second forced break (longer, if the observer wanted). Each observer completed a practice trial followed by a single block with six trials.

#### Structure-From-Motion

This stimulus was very similar to the one used in a previous study (Brascamp et al., 2018). It consisted of horizontally moving dots (60 cd/m^2^, radius 0.05 dva) with a sinusoidal speed profile across space, presented on a gray background (20 cd/m^2^; schematically represented in Figure 2). The only difference between the stimulus used in this study and the one used in the previous study is that each dot had a lifetime of 2 seconds after which it was replaced by another dot at a random location. This motion sequence was perceived as an orthographic projection of a transparent, rotating, sphere with a radius of 2 dva. The observers were asked to fixate on a round fixation mark at the center of the display (1 cd/m^2^; radius 0.05 dva) and to indicate the first rotation direction they perceived as well as any change in this perceived direction (clockwise, counterclockwise, or a nonexclusive mixture of these percepts) using three separate keys on the keyboard. Each observer completed one block which consisted of a practice trial of 45 seconds (data not analyzed) in the beginning followed by ten 60 second trials where each pair of trials was separated by a forced break of a minimum of 10 seconds long.

#### Center-Surround Stimulus

**Figure 3.**
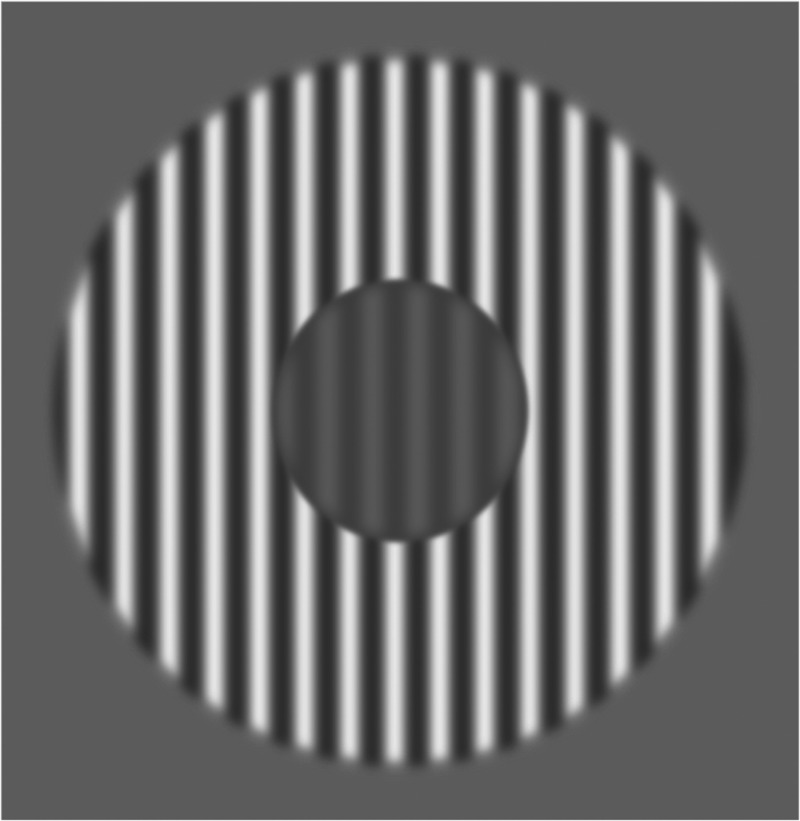
Center-surround suppression stimulus. This is a schematic representation of the center-surround test stimulus. The participants were asked to compare the contrast of the central part of this stimulus (while ignoring the surround grating) with a reference stimulus which did not have a surround. The contrast of the central area of the test stimulus was kept constant at 0.2, whereas the surround had a contrast of 0.8. The contrast of the reference stimulus was varied from trial to trial.

This task was derived from Xing and Heeger (2000). It involved a circular central grating disk (spatial frequency of 2 c/d) and a surrounding grating annulus (same spatial frequency; Figure 3). The contrast of the surrounding annulus was 0.8 (Michelson), and the contrast of the central disk was 0.2. This type of stimulus usually induces perceptual suppression, in the sense that the contrast of the center is typically underestimated for this type of stimulus. Perceptual suppression in such paradigms is often thought to be associated with cortical inhibition (Angelucci et al., 2017; Flevaris & Murray, 2015; Robertson et al., 2016; Serrano-Pedraza et al., 2014; Spiegel et al., 2019; Tibber et al., 2013; Yoon et al., 2010). Each trial consisted of two center grating disks presented sequentially at fixation for 0.5 seconds each, separated by a 0.3-second blank. The observer was asked to report which of the two gratings had a higher contrast. One of the central disks served as a reference stimulus and was presented without a surround. The contrast of this reference stimulus varied from trial to trial. The other central disk of the trial was the actual test stimulus; it always had the same contrast and it was accompanied by a surround. The order of the test stimulus and reference stimulus was assigned randomly on each trial. The orientation of both the center gratings and the surround gratings was vertical, and there was a 180° phase difference between the two gratings at all times. The phase of all gratings shifted by 180° at a rate of 8 times per second during each presentation. The center stimulus had a radius of 1.75 dva, the inner radius of the surround stimulus was identical to the outer radius of the center stimulus, and the outer radius of the surround stimulus was 5 dva. The outermost part of the center stimulus (0.2 dva) was occupied by a raised cosine edge. Similarly, the innermost part (0.2 dva) of the surround stimulus was occupied by such an edge.

Each observer ran a single block involving this stimulus, which consisted of three interleaved one-up-one-down staircases during which the reference contrast was varied. For the three staircases, the reference contrast started, respectively, at 75%, 100% or 125% of the test contrast for that condition. Each staircase had 40 trials. After each trial, the reference contrast was incremented or decremented (depending on the response) by a set amount. At the beginning of a given staircase, this set amount was 50% of the current reference contrast. Every time a reversal occurred within a staircase (i.e., the observer said “reference contrast is higher” on the previous trial and “test contrast is higher” on the current trial, or vice versa), the current percentage amount was multiplied by 0.8, but it was not allowed to drop below 5%. The perceived contrast of the test stimulus was calculated by coding the *reference is higher* and *test is higher* responses as 1 and 0, respectively, and plotting these responses for all trial as a function of the reference contrasts on all those trials. The perceived contrast of the test stimulus was then computed as the mean of a cumulative Gaussian fitted to this relationship. Aside from the mean and standard deviation (*SD*), there was a third free parameter (a “lapse rate” parameter) that we used for fitting the cumulative Gaussian. This parameter scaled the vertical range of the function so that it could be smaller than the usual range of 0 to 1. This was done keeping in mind that motor errors or attention lapses may prevent the curve from spanning the full range from 0 to 1, as those might cause a nonzero error rate even on trials where the difference in contrast between the test stimulus and the reference stimulus is easy to perceive.

Each observer had 10 practice trials before the beginning of the experiment block. Observers were asked to fixate at a black round fixation point (radius 0.5°) throughout the stimulus presentation periods and the interstimulus intervals. They had to report which contrast (first or second stimulus) appeared higher using two keys on the keyboard.

### Data Analysis

Percept durations were calculated by taking the time interval separating the start of one exclusive percept from the start of the following alternative exclusive percept, so including any intervening mixture periods (i.e., “start-to-start” durations; Brascamp et al., 2018). For each of the bistable stimuli used here (binocular rivalry, bistable moving plaids, and structure-from-motion), these percept durations were subsequently averaged within participants and then log-transformed to convert the right-skewed percept duration distributions to ones that more closely approximate normality.

As soon as the data were collected, they were subjected to preliminary analysis to be sure that there were no issues that would prevent interpretation of our main analyses. In the case of the bistable perception paradigms, the data were excluded if the participant reported fewer than two perceptual alternations for a given paradigm in total. For binocular rivalry and structure-from-motion, participants were also excluded from analysis if they did not meet the following “balance criterion”: The total amount of time one percept was reported needed to be less than 3 times the total amount of time the other percept was reported (corresponding to a balance between percepts less extreme than 75%/25%). This exclusion criterion was not applied to the bistable moving plaid data because the percepts of the bistable plaid stimulus are inherently asymmetrical. For the structure-from-motion stimulus and the bistable plaid stimulus, participants were also excluded if they reported a mixture percept for more than 50% of the viewing time. This cutoff was a little higher, 60%, for binocular rivalry as the tendency to perceive a mixture percept is much higher for binocular rivalry than for the other two paradigms. An additional balance criterion was also applied to all the bistability paradigms such that no more than 50% of reported perceptual transitions were allowed to be return transitions. A return transition is an instance when the participant reports a change in perception from an exclusive percept to a mixture percept and back to the same exclusive percept (Mueller & Blake, 1989). For the center-surround suppression task, we applied an exclusion criterion based on the slope of the psychometric curve that relates the probability of a “reference contrast higher” response to the physical contrast of the reference stimulus. A shallow slope here indicates that a participant’s responses regarding the reference contrast show little dependence on the physical value of that contrast, suggesting that the participant did not follow the instructions. Because curve slope in this case depended on the combination of two parameters (both the *SD* and the lapse rate), we quantified slope as the difference in the curve’s vertical position between the two horizontal points located at 5% contrast to the right of the curve mean and 5% contrast to the left of the curve mean. Participants whose average curve slope across this 10% contrast interval was less than 2 were excluded from the main analysis (25 participants were excluded as a result of this criterion). In other words, only the participants who had at least a 20% change in their probability of judging the reference contrast as higher than the test contrast across this range of reference contrast values around the curve mean were included for further analysis. In addition, we excluded a handful of participants whose curve slopes indicated they were judging the reference contrast, yet for whom the curve mean (i.e., the reference contrast value that perceptually matches the test contrast) was unusually high. In particular, whereas the test contrast was 0.2, a few observers had curve means that were closer to the contrast of the surround of the test stimulus, which was 0.8. We suspect that those participants misunderstood the task and compared the contrast of the reference grating with the contrast of the surround grating instead of that of the center, test, grating. Four participants were excluded on these grounds, all of whom had curve means higher than 0.65.

To compute reliability for the bistable perception paradigms, we calculated the correlation between two randomly selected, nonoverlapping, halves of all collected percept durations for each observer. We repeated this a 1,000 times, each time using a different random selection, and obtained the mean correlation coefficient across these thousand runs. This value was then corrected using the Spearman–Brown prediction formula. For the center-surround suppression strength measure, we calculated the correlation between the odd and the even trials of each condition and then again used the Spearman–Brown prediction formula to extrapolate the observed correlation coefficient to the one predicted for twice the amount of data.

Finally, Pearson correlations were calculated for all combinations of the paradigms. In addition, a mediation analysis was carried out to determine whether surround suppression could be the mediating factor that explains the correlation between binocular rivalry and bistable moving plaids. In other words, this latter analysis tested whether between-observer variability in surround suppression strength was correlated with between-observer variability that is shared between the binocular rivalry paradigm and the bistable moving plaids paradigm.

## Results

**Figure 4.**
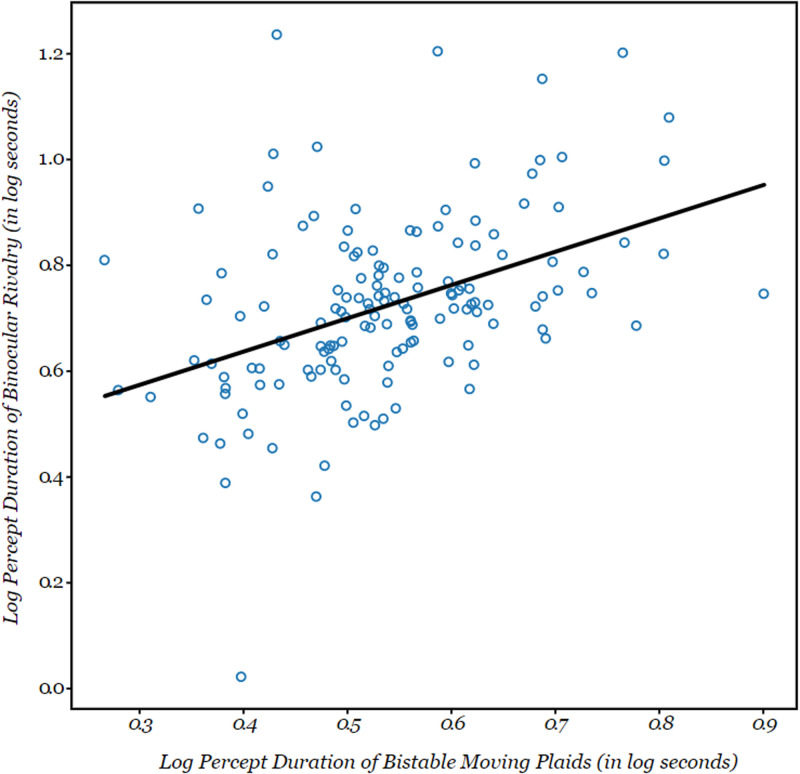
Plot showing a positive correlation between the percept durations for the bistable moving plaid paradigm and binocular rivalry paradigm, *r*(163) = .445; *p* < .001. Each individual circle corresponds to an individual participant.

**Figure 5.**
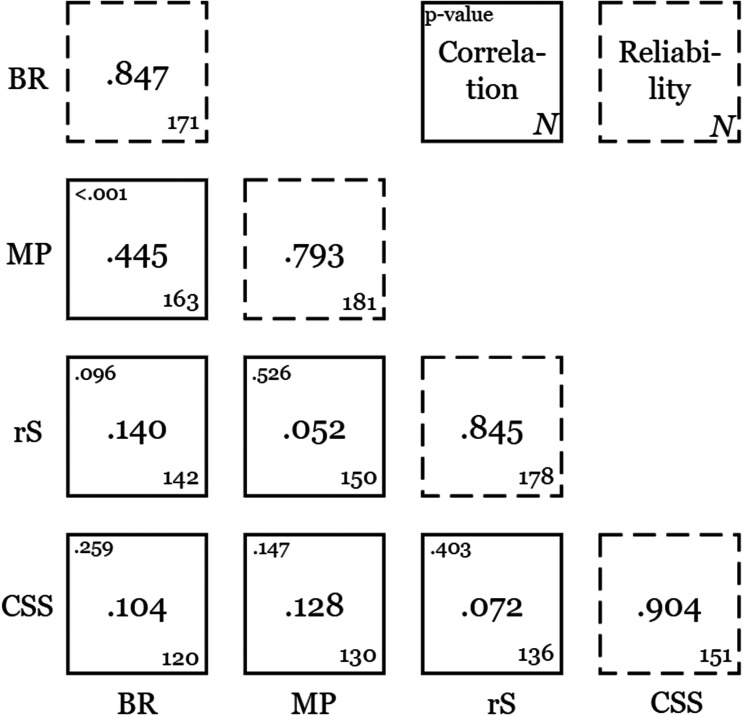
Correlation matrix of the various bistable perceptual paradigms used in this study. Solid squares represent correlations between paradigms, and broken squares represent reliabilities associated with individual paradigms. BR = binocular rivalry; MP = bistable moving plaids; rS = bistable structure-from-motion (rotating sphere); CSS = center-surround suppression. The number at the bottom right of each of the squares represents the N for the given paradigm or pair of paradigms.

We first performed preliminary analyses on the data from the bistable perception paradigms by way of sanity check. We analyzed the reliability of each bistable perception paradigm as indicated in “Data Analysis” section and found high reliabilities associated with all the paradigms (Figure 5). We also investigated whether we could replicate the previously reported across-observer correlation between the average percept duration of binocular rivalry and of bistable moving plaid perception as well as their potential correlations with average percept duration of the rotating sphere. As shown in Figure 4, the results from this correlation analysis indicate that the log-transformed percept durations of binocular rivalry and bistable moving plaids were positively correlated, *r*(163) = .445; *p* < .001; 90% confidence interval [CI] = [0.32, 0.57]. This correlation replicates earlier work and supports the notion that there is a shared mechanism between these two bistable perception paradigms. The (log-transformed) percept durations of the bistable structure-from-motion stimulus, on the other hand, did not significantly correlate with those for either binocular rivalry, *r*(142) = .14; *p* = .095; 90% CI [−0.02, 0.30], or the bistable moving plaids, *r*(150) = .052; *p* = .526; 90% CI [−0.11, 0.21], which matches the fact that reports of a presence of these correlations in the literature have been less unanimous.

Before continuing on to the main analyses, we also performed preliminary analyses of the center-surround data to evaluate their robustness. First, we calculated the reliability for the center-surround paradigm, as we did for the bistability paradigms, and indeed, we found this paradigm to be associated with high reliability, as indicated in Figure 5. Next, given that a center-surround stimulus like ours typically results in perceptual suppression, we expected that same general pattern in our data. Indeed, the perceived contrast for the center stimulus, *M*(151) = 0.178, *SD* = 0.08, was significantly lower than its physical contrast of 0.2, *t*(151) = −3.342, *p* = .001. This argues in favor of the, for our purposes, critical presence of perceptual suppression in our center-surround paradigm.

**Figure 6.**
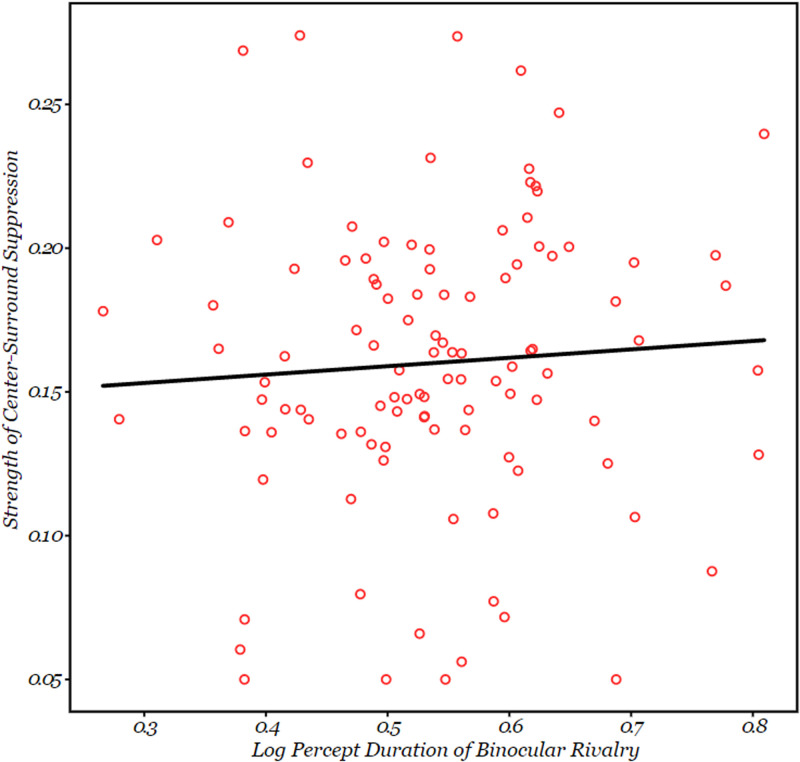
Plot showing a correlation between the percept durations for binocular rivalry and strength of surround suppression, r(120) = .104, p = .259. Each individual circle corresponds to an individual participant.

Together, these results provide the necessary preconditions for our main analysis of correlations involving both bistable perception and surround suppression, and the good match with existing literature instills confidence in the sanity of our data.

As stated in “Introduction” section, we hypothesized that the correlation between perceptual switch rates in binocular rivalry and bistable plaid perception could be accounted for, in part, by variance in the perceptual strength of center-surround suppression. Such a data pattern would support the notion that cortical inhibition, as indexed by center-surround suppression, is a factor that underlies the correlated percept durations of binocular rivalry and the bistable moving plaid stimulus. To test our hypothesis, we first analyzed the correlations between percept durations for each bistable perception condition individually on the one hand, and the strength of center-surround suppression on the other, all using the Pearson correlation coefficient. The analysis revealed that there was no significant correlation between the (log-transformed) percept durations of binocular rivalry and surround suppression strength, *r*(120) = .104, *p* = .259; 90% CI [−0.07, 0.28], shown in Figure 6. Similarly, for the log-transformed average percept durations for the bistable moving plaid the correlation with surround suppression strength was not significant, *r*(130) = .128, *p* = .147; 90% CI [−0.04, 0.30]. We also found no significant correlation between structure-from-motion percept duration (log-transformed) and perceived contrast in the surround suppression condition, *r*(136) = .072, *p* = .403; 90% CI [−0.10, 0.24]. The correlations among all paradigms are tabulated in Figure 5, which also indicates the reliabilities of each of the paradigms.

**Figure 7.**
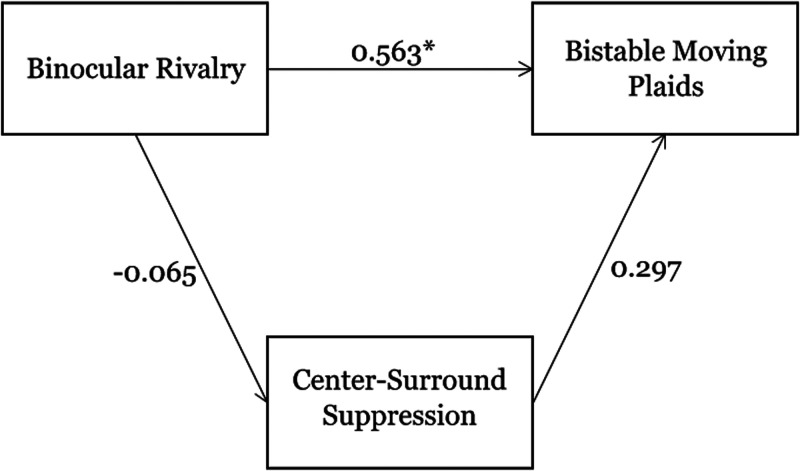
Mediation analysis. Here, we treated (log-transformed) average percept durations for binocular rivalry and for bistable moving plaids as the independent variable and dependent variable, respectively, while treating center-surround suppression strength as the mediator. We observed a significant direct effect and an indirect effect that was small and not significant, indicating that across-observer variance in center-surround suppression strength cannot explain the correlation between the dynamics of binocular rivalry and bistable plaid perception.

The lack of significant correlations in our data between center-surround suppression strength and the percept durations for binocular rivalry or bistable moving plaids argues against the idea that the observed correlation between the latter two variables can be explained by the former variable. Still, to formally test this notion, we performed a mediation analysis on our data. Here, we treated (log-transformed) average percept durations for binocular rivalry and for bistable moving plaids as the independent variable and dependent variable, respectively, while treating center-surround suppression strength as the mediator. This analysis, in other words, tests whether interobserver differences in the binocular rivalry data are predictive of interobserver differences in the bistable moving plaid data, and whether any of that predictiveness is captured by interobserver differences in the center-surround data. Consistent with the simple correlation analyses reported earlier, the total effect was strong and significant (coefficient = .576; *p* < .001). This number quantifies the overall relation between the independent variable and dependent variable, irrespective of the mediator. Also consistent with those correlation analyses, the standardized regression coefficients for binocular rivalry percept durations versus surround suppression strength and for bistable moving plaid percept durations versus surround suppression strength were not significant (coefficient = − .065; *p* = .272, and coefficient = .297; *p* = .108, respectively, shown in Figure 7). The key question, then, is whether any part of the total effect reflects an indirect effect involving the mediator, that is, the center-surround data. Instead, the indirect effect had a coefficient of .013 and was statistically not significant (*p* = .528 and 90% CI [0.005, 0.105]), and the overall effect was essentially completely due to a direct effect not involving the mediator (coefficient = .563, *p* < .001, shown in Figure 7). Thus, the results from this mediation analysis, along with the above-reported correlation results, indicate that variance in center-surround suppression strength cannot explain the correlation between the dynamics of binocular rivalry and of bistable plaid perception.

## Discussion

In this study, we successfully replicated the correlation in average percept duration between binocular rivalry and bistable moving plaids. This correlation indicates that there is an overlap between the mechanisms that drive perception in these two paradigms. The lack of correlation between percept durations in these paradigms and various other bistable perception paradigms (Brascamp et al., 2018; Cao et al., 2018; Gallagher & Arnold, 2014), including structure-from-motion in this study, argues against a global shared mechanism such as degree of attention to the task (although see Pastukhov et al., 2019). We hypothesized that the strength of cortical inhibition could be the common underlying factor that accounts for the correlated perceptual dynamics of binocular rivalry and bistable moving plaids. We assessed the perceptual strength of center-surround suppression as an index for cortical inhibition. Although our analyses indicated that our measures of perceptual bistability and of center-surround suppression were reliable, we did not find a correlation between the two types of measures. The lack of correlations of the percept durations of binocular rivalry and bistable moving plaid perception with the strength of center-surround suppression, along with the lack of an indirect effect in the mediation analysis, do not support the idea of cortical inhibition as a shared factor that can explain the correlated dynamics of binocular rivalry and bistable moving plaid perception.

Although we observed significant surround suppression, the magnitude of this suppression does not quite match the magnitude of this variable as observed in some other studies. We used our data to calculate the suppression ratio, which is defined as the matching contrast divided by the test contrast. A ratio of 1 indicates veridical perception, whereas suppression and facilitation are indicated by values of less than 1 and more than 1, respectively. The suppression ratio in our study was found to be 0.89. This indicates much weaker suppression than what Xing and Heeger (2001) found in a seminal study, as they reported a suppression ratio between 0.5 and 0.6 for an identical stimulus. One possible reason for the lower degree of suppression in our study relates to the fact that our observers were 151 untrained undergraduate students who completed a battery of many different types of experiments, whereas only 2 participants (one of them being an author) were included in the Xing and Heeger’s study, and both collected data across a large number of conditions. It is possible, therefore, that the difference in suppression ratio indicates a relatively large amount of noise in our data, stemming from a relative lack of expertise and motivation, on average, in our participant sample. Consistent with this notion are the findings from another study (Karas & McKendrick, 2015) that involved 30 participants. Both this sample size and the amount of data per participant suggest a level of expertise on the part of the participants that is intermediate between the levels in our own study and in the one by Xing and Heeger (2001). Consistent with an account in terms of expertise (and perhaps motivation), the suppression ratios in that study are consistently higher (indicating weaker suppression) than those from Xing and Heeger across a range of conditions, although still somewhat lower than ours in a condition that matches our own (between approximately 0.7 and 0.75). In sum, the evidence from this study reinforces the notion that our participants’ relative lack of expertise might explain the relatively weak surround suppression in our study. This raises the possibility that, whereas we did not find any correlation between surround suppression strength and bistable perception dynamics, such a correlation may have been observed if only we had observed stronger surround suppression. To examine this idea, we further analyzed the data by dividing our sample into two halves based on the participants’ performance on the center-surround suppression task, such that one half consisted of the top 50% of participants who show the strongest center-surround suppression, and the other half consisted of the remaining participants. Unsurprisingly, for the group with strong center-surround suppression, the perceived contrast of the center stimulus reduced, from *M*(151) = 0.178, *SD* = 0.08 to *M*(75) = 0.133, *SD* = 0.028, thereby improving the suppression ratio to 0.67; comparable to the values observed in the two studies mentioned earlier. More interestingly, the correlation between binocular rivalry and moving plaids also becomes stronger for this group, going from *r*(163) = .445, *p* < .001, 90% CI [0.32, 0.57] to *r*(58) = .582, *p* < .001, 90% CI [0.49, 0.84]. This reinforces the idea that, by selecting this half of the observers we are focusing on participants with relatively noise-free data. In agreement with this observation, the remaining 50% of observers, the ones with weak center-surround suppression, show a weaker, but still significant, correlation between the perceptual dynamics of binocular rivalry and bistable moving plaid, *r*(57) = .383, *p* = .003, 90% CI [0.14, 0.67], and for the center-surround suppression task, these remaining observers also have an interobserver spread in matching contrast that is larger than the corresponding value for the entire group of observers, *M*(74) = 0.254, *SD* = 0.14, versus *M*(151) = 0.178, *SD* = 0.08 for the entire group, even though this subset has been specifically selected for having similar matching contrasts (i.e., the 50% least suppressed matching contrasts). In other words, the remaining 50% of observers who are weeded out by focusing on the 50% who have strong center-surround suppression show all signs of having relatively noisy data. Now the critical question becomes, if we focus on the 50% of observers who have strong surround suppression and whose data suggest a relative lack of noise as compared with the rest of our sample, do we observe a correlation between center-surround suppression strength and bistable perception dynamics? The answer is no. Those correlations remain weak and statistically nonsignificant for the 50% of the observers who have strong surround suppression, center-surround suppression versus binocular rivalry: *r*(59) = −.078, *p* = .558, 90% CI [−0.33, 0.18]; versus bistable moving plaids: *r*(64) = −.025, *p* = .847, 90% CI [−0.27, 0.22]; and versus structure-from-motion: *r*(64) = −.039, *p* = .76, 90% CI [−0.29, 0.21]. Therefore, it would be reasonable to conclude that the lower strength of suppression in our study does not explain the lack of correlation of center-surround suppression strength with perception in our bistable perception paradigms.

This study relies on the assumption that center-surround suppression is a good behavioral index of cortical inhibition. Whereas our data provide a clear indication that the strength of center-surround suppression—a behavioral index—does not explain substantial variance in the dynamics of bistable perception, we can be less certain about the strength of cortical inhibition—a neural measure. To what extent does center-surround suppression serve as a reliable index for cortical inhibition? There have been numerous studies that explored this idea. Indirect evidence for the validity of various forms of center-surround suppression as an index of cortical inhibition comes from work involving special populations. For example, in their study involving older adults, Pitchaimuthu et al. (2017) not only report that the concentration of GABA is elevated in the visual cortex of older adults, but also that this higher concentration of GABA was associated with reduced motion suppression indices. Multiple other studies have linked disorders like schizophrenia and ASD to cortical excitatory–inhibitory imbalance and have also reported that these populations perform differently to various perceptual surround suppression paradigms as compared with the healthy population, although it should be noted that there are a number of center-surround paradigms that were used in these studies (Flevaris & Murray, 2015; Robertson et al., 2016; Serrano-Pedraza et al., 2014; Spiegel et al., 2019; Tibber et al., 2013; Yoon et al., 2010). In addition, numerous neurophysiological studies, as reported in Angelucci et al. (2017), have proposed that neural inhibition is involved in center-surround suppression although they suggest that suppression can also result from other factors, including increased bottom-up inhibitory input and decreased excitatory inputs in the local recurrent connections in the visual cortex. In sum, there is good evidence of a relation between perceptual center-surround suppression and cortical inhibition. So in that light a lack of correlation, in the present work, between interindividual differences in center-surround suppression strength and in measures of bistable perception, really would argue against a key role of cortical inhibition in driving interindividual differences in bistable perception.

How do our results compare to existing work that explores the relationship between the dynamics of bistable perception and the strength of cortical inhibition? Various studies on disorders like schizophrenia and ASD have linked disturbances in the excitatory–inhibitory balance in visual cortex with altered dynamics of bistable perception (Robertson et al., 2016; Spiegel et al., 2019; Yoon et al., 2010). In addition, a study on older adults has reported a modest correlation between the concentration of GABA in visual cortex and perceptual dynamics of binocular rivalry (Pitchaimuthu et al., 2017). On the other hand, a study investigating the excitatory–inhibitory neurochemicals in migraineurs and healthy controls found no overall correlation between the concentration GABA in visual cortex and perceptual dynamics of binocular rivalry (Chan et al., 2019). There are also two behavioral studies, unrelated to disorders, that report an across-observer correlation between binocular rivalry percept durations and the strength of center-surround suppression (McKendrick et al., 2019; Steinwurzel et al., 2020), in contrast to our present findings. Steinwurtzel et al. used a tilt illusion, rather than a contrast illusion, to index center-surround suppression, providing one potential explanation for the difference in results. McKendrick et al. (2019) did use a contrast illusion like ours as their measure of center-surround suppression, but it should be mentioned that theirs was a preliminary study. Specifics of experimental paradigm aside, both Steinwurzel et al. (2020) and McKendrick et al. (2019) interpreted their results in terms of the role of cortical inhibition in binocular rivalry. In the face of the combined evidence from all available studies, as well as the intuitive appeal of the notion that cortical inhibition is involved in perceptual suppression, it would not be warranted to use our results as a basis to strongly oppose that notion. However, our results do argue against the idea that interobserver variability in the strength of cortical inhibition, to the extent that it is expressed in the perceptual strength of center-surround suppression, can account for interobserver variability in the dynamics of bistable perception.

There are several possible explanations as to why we did not replicate the previous findings of an interobserver correlation between binocular rivalry measures and surround suppression measures. As already briefly mentioned earlier, there are differences in the particulars of the stimuli and the paradigms used in the earlier studies (McKendrick et al., 2019; Steinwurzel et al., 2020) as compared with our study. Aside from stimulus differences, it is possible that the correlations between binocular rivalry perception and center-surround suppression that were reported in previous work were false positives, or that the lack of any such correlation in our present work amounts to a false negative. Therefore, it is relevant to compare indices of statistical power between studies: The number of participants in our study (i.e., 120 included for both binocular rivalry and center-surround suppression, after applying exclusion criteria) is considerably larger than the numbers from the previous studies (50 and 57 for Steinwurtzel et al. and McKendrick et al., respectively). When it comes to the total duration of viewing, which in the Steinwurzel et al.’s (2020) study was also reported, our binocular rivalry task consisted of six trials that lasted for a combined duration of 4 minutes per observer (excluding the mimic condition) as compared with two trials lasting for a combined duration of 6 minutes for Steinwurzel et al. (2020), suggesting that per-observer estimates of percept duration were slightly more reliable in that existing study.

What can be concluded about the relationship between the dynamics of structure-from-motion perception with the dynamics of both binocular rivalry and bistable moving plaid perception? Our data shed some light on a potential correlation between the percept durations of binocular rivalry and structure-from-motion, which has been reported by one previous study (Steinwurzel et al., 2020), but which was not observed by others (Brascamp et al., 2018; Cao et al., 2018). Our data are consistent with the idea that there might be a very modest and fragile correlation between perception of the structure-from-motion stimulus and that of both binocular rivalry and bistable moving plaids. Above we reported that we found no correlation between structure-from-motion and either binocular rivalry or bistable moving plaids in the present study, which is true when using the Pearson correlation coefficient, as we had set out to do at the start. An exploratory analysis using Spearman correlation coefficients instead, however, indicates a weak correlation between the (log-transformed) percept durations of structure-from-motion perception and binocular rivalry, ρ(142) = .213, *p* = .011, although not between structure-from-motion perception and bistable moving plaids, ρ(150) = .095, *p* = .247. In addition, when we analyzed the data by dividing our sample into two halves based on the participants’ performance on the center-surround suppression task, as described earlier, we found that the Spearman correlations with structure-from-motion became statistically significant for both binocular rivalry, ρ(52) = .358, *p* = .009, and bistable moving plaids, ρ(56) = .263, *p* = .05, although Pearson correlations remained nonsignificant. In light of this ambiguity in our own data, as well as the conflicting results in the literature, it seems plausible that there is some correlation between the percept durations during structure-from-motion perception and those during binocular rivalry or bistable moving plaid perception, but that it is not sufficiently strong to be observed consistently.

As mentioned in “Introduction” section, this work is partly inspired by previous work that showed correlations between distinct bistable stimuli in terms of the temporal dynamics of the perception they elicit. Such correlations can be informative as to what drives those dynamics. Our present findings add to the body of work showing that some bistable stimuli do correlate in that regard, whereas others seem not to. Across the available papers, the most consistently reported correlation seems to be the one between the percept durations of binocular rivalry and those of bistable moving plaids (Brascamp et al., 2019; Cao et al., 2018; Sheppard & Pettigrew, 2006), and our present results provide further confidence that that correlation is robust. Correlations involving other bistable stimuli are often not observed. From the perspective that between-paradigm correlations may inform about underlying mechanisms, the notion that correlations are strong between only some bistable perception paradigms yet weak or absent between others, renders the more consistent correlations all the more interesting: Apparently, they do not reflect nonspecific factors that influence perceptual bistability across the board.

In sum, our findings add to the body of work that delineates which bistable perception paradigms do correlate with each other in terms of perceptual dynamics and which do not, and our findings do not provide support for the idea that those correlations that do exist, reflect interobserver variability in the strength of cortical inhibition.
